# Resection of colorectal liver metastases following neoadjuvant chemotherapy

**DOI:** 10.3332/ecancer.2008.58

**Published:** 2007-10-16

**Authors:** A Chiappa, E Bertani, R Biffi, U Pace, G Viale, G Pruneri, G Zampino, N Fazio, F Orsi, G Bonomo, L Monfardini, P Della Vigna, B Andreoni

**Affiliations:** 1 Department of General Surgery-Laparoscopic Surgery, European Institute of Oncology, University of Milano, 20141, Italy; 2 Division of Abdomino-pelvic Surgery, European Institute of Oncology, University of Milano, 20141, Italy; 3 Department of Pathology, European Institute of Oncology, University of Milano, 20141, Italy; 4 Department of Oncology, European Institute of Oncology, University of Milano, 20141, Italy; 5 Department of Radiology, European Institute of Oncology, University of Milano, 20141, Italy

## Abstract

**Background/aims:**

Hepatic resection in metastatic disease from colorectal cancer offers the best chance in selected cases for long-term survival. Neoadjuvant chemotherapy (NACT) has been advocated in some cases initially deemed irresectable, with few reports of the efficacy of such a strategy and the influence of the response to chemotherapy on the outcome of radical hepatic resection.

**Methodology:**

Between December 1995 and May 2005, 27 patients with colorectal liver metastases (seven males, 20 females, mean age: 58 ± 8 years; range: 40–75) were treated with neoadjuvant chemotherapy. A seven-year survival analysis was performed. Chemotherapy included mainly 5-fluorouracil, leucovorin and either oxaliplatin or irinotecan for a median of eight courses.

**Results:**

A total of 16 patients (59%) had synchronous and 11 (41%) metachronous metastases. During pre-operative chemotherapy, tumour regression occurred in ten cases (37%), stable disease in a further ten patients (37%) and progressive disease developed in seven cases (26%). The five-year overall survival for NACT responders was 64% and only 15% for non-responders (p=0.044).

**Conclusions:**

The response to chemotherapy is likely to be a significant prognostic factor affecting survival after liver resection for cure.

## Introduction

Surgical resection of colorectal liver metastases, where possible, still remains the only treatment ensuring long-term survival, when margin-free (R0) hepatic resection can result in 25–35% reported overall five-year survival [1–7]. This contrasts with the dismal long-term prognosis either of untreated hepatic metastases [8] or even that where conventional chemotherapy alone is used [9–11]. Controversy still remains concerning the timing of hepatectomy in resectable cases, either as a stage delayed or as a synchronous resection [12, 13] as well as the neoadjuvant usage of chemotherapy prior to hepatic resection particularly if the disease is initially assessed as irresectable [14, 15].

The perioperative risks of liver resection have been substantially reduced in recent years. It is not, however, clear whether systemic pre-operative chemotherapy increases the morbidity of subsequent surgery through the induction of steatosis, sinusoidal congestion and centrilobular necrosis as has been reported with fluoropyrimidine analogues [16, 17] or as a result of an increased risk of bleeding during surgery where the post-chemotherapy hepatic parenchyma tends to be more congested and friable following intra-arterial therapy [18]. The effects of systemic chemotherapy on the liver parenchyma and on the post-operative course following resection have been relatively poorly assessed in groups of patients where there has been considerable heterogeneity of chemotherapeutic agents utilized and variation in the types and techniques of liver resections performed [19–22]. The present study reports the effects and outcome of pre-operative systemic chemotherapy use in a consecutive series of 27 patients presenting with colorectal liver metastases as an aid and a guide to definitive metastasis resection with curative intent.

## Methodology

Between April 1996 and February 2005, 27 consecutive patients affected by colorectal liver metastases received systemic chemotherapy prior to liver surgery (seven males; 20 females; mean overall age: 58 ± 8 years; range: 40–75 years). The rationale of this approach relies on the assumption that occult micro-metastases may be present and that neoadjuvant chemotherapy is likely to improve the performance of a radical (R0) liver resection and that metastases initially deemed irresectable may be rendered operable for resection with curative intent.

### Patients’ characteristics

The relevant clinical data and tumour characteristics are shown in Table 1. Liver metastases were synchronous in 16 cases (59%) and bilobar in four cases (15%). The median maximal diameter of the metastases was 15 mm (range: 5–80 mm). Extrahepatic metastases were detected pre-operatively in three patients (11%) with all extrahepatic sites being technically resectable either sequentially or at the time of the initial liver resection. The sites of the extrahepatic tumours were either lung (two cases) or the site of the original primary tumour resection (one case).

### Neoadjuvant chemotherapy

The objectives in medical management were different according to the initial resectability of the metastases. For unresectable patients, chemotherapy was the only means to convert cases into a resectable state and was utilized for longer. For resectable patients, the first objective of the chemotherapy was to provide a time interval before surgery for assessment of the tumour biology, to treat potentially occult disease and to avoid surgery in those patients with rapidly progressive disease as a result of primary resistance to chemotherapy. A second objective in these resectable patients was to achieve cytoreduction both to limit the extent of liver resection and potentially post-operative morbidity as well as to facilitate a margin-free R0 liver resection. The median number of cycles of pre-operative chemotherapy per patient was eight (range: 2–12). In ten patients, systemic chemotherapy was continued post-operatively for a median of six cycles.

The response to chemotherapy was evaluated from serial imaging studies (thoraco- and abdomino-pelvic CT scan or MRI scanning of the abdomen where indicated) and was based on the change in tumour diameter according to the World Health Organisation criteria [23]. Response was defined as a 50% or more decrease in the total tumour size of lesions, with stabilization being defined as a less than 50% decrease or a less than 25% increase in the total tumour size. Progression was classified as a 25% or more increase in the total tumour size and/or the appearance of new lesions at any site. When more than one treatment regimen was used in the same patient, the response to the last regimen used pre-operatively was considered for analysis.

### Selection for liver resection

Patients were eligible for hepatic resection when the following conditions were met, namely: (1) no co-morbid conditions were present precluding liver resection, (2) all malignant liver disease was amenable to resection and ablative treatment whilst being able to retain at least 30% of non-tumoural liver parenchyma, (3) recurrence of the primary tumour was excluded and (4) either no non-resectable extrahepatic disease was detected by pre-operative serial imaging studies, or where potentially resectable extrahepatic tumour was detected, it was not considered a contraindication to sequential or synchronous surgery. The time interval between the final chemotherapy dose and hepatic surgery was usually 2–4 weeks to minimize the risk of tumour progression and to reduce perioperative morbidity. This approach was adopted in accordance with other similar reports [24, 25]. The policy of liver resection attempted a radical resection either by anatomic or non-anatomic (wedge) resection, sparing the largest amount of liver parenchyma possible but providing a tumour-free margin of 1 cm whenever feasible. All procedures routinely used intra-operative ultrasound (IOUS), an ultrasonic dissector for parenchymal transection (CUSA) and a combination of the argon beam and bipolar coagulation forceps to reduce intra-operative blood loss. The radio-frequency ablation device (TARF) was used in combination with conventional surgery to treat non-resectable remnant lesions, thus permitting an extension of the indications for liver resection in patients who otherwise would not have been candidates for surgery.

### Post-operative follow-up

Patients were followed up one month after surgery and then every four months thereafter with evaluation of tumour markers (CEA, and CA 19-9 serum levels), liver function tests and by hepatic ultrasound. An abdomino-pelvic or thoraco-abdominal CT scan was performed every six months during the follow-up. In the case of resectable extrahepatic metastasis(es), sites were resected 203 months following definitive hepatic surgery, using systemic chemotherapy between operations in order to prevent tumour progression.

## Analysis of the data

Overall and disease-free survival probabilities were determined by the Kaplan-Meier analysis [26] and compared using the log-rank test [27]. A multivariate analysis using a Cox model was performed to determine independent prognostic factors for survival, with p values < 0.05 being considered significant.

## Results

Liver resection was performed following an objective tumour response in ten patients (37%), after stabilization in a further ten patients (37%) and after tumour progression in seven patients (26%). Major liver resections (≥ 2 segments). were all performed with curative intent and included two right hepatectomies, one left hepatectomy, one extended right hepatectomy, four bisegmentectomies, two bisegmentectomies plus one segmetectomy and two bisegmentectomies plus a wedge resection. For two patients (3%), liver resection was combined with a gastrointestinal resection (namely an anterior resection of the rectum). Perioperative mortality was nil (30 days following surgery). Eighteen patients (67%) were discharged without complications with one patient (3%) undergoing a right hepatectomy having mild post-operative reversible liver failure. Among the minor complications in nine patients (33%), there were three pleural effusions, two abdominal collections, two wound infections and two central venous catheter (CVC) infections. All patients underwent hepatic resections with curative intent (R0 resections) with no evidence of microscopic involvement of the surgical margin. The patient and tumour characteristics according to response to pre-operative chemotherapy regimens utilized are shown in Table 2.

### Outcome

Following a mean follow-up of 36 months (range: 8–110 months), 18 patients suffered from recurrence (67%) amongst the 27 patients. Liver recurrence was isolated in eight patients (30%) and associated with extrahepatic recurrence in four cases (15%). Of the 12 patients with hepatic recurrence, two underwent a repeat hepatectomy. Of the 25 patients initially free of extrahepatic disease, ten subsequently developed extrahepatic recurrences (37%). Of these latter four patients underwent one or more re-operations for extrahepatic recurrence.

### Survival

**Figure 1:**
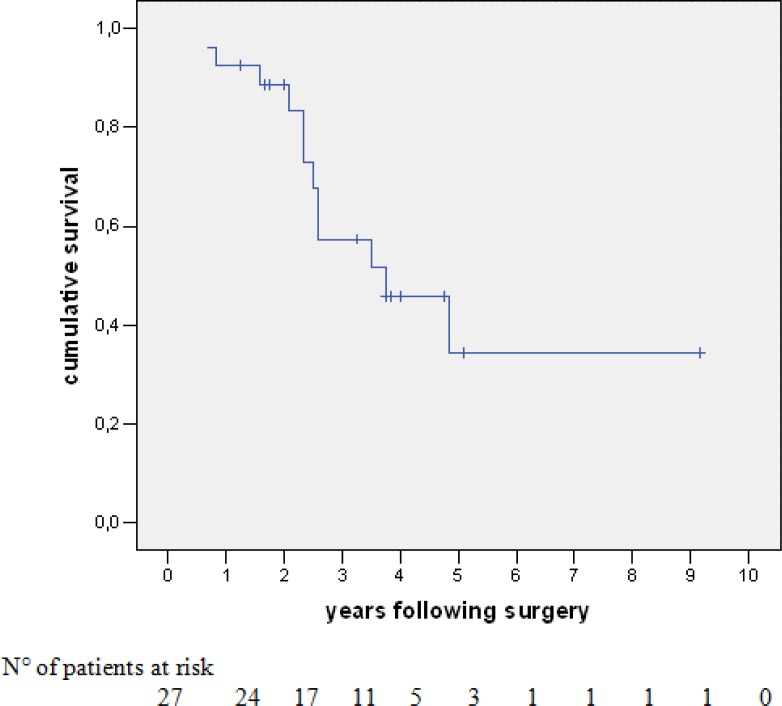
Survival of 27 patients undergoing liver resection following neoadjuvant chemotherapy for colorectal-cancer liver metastases.

The overall survival (OS) of the 27 patients was 93%, 57% and 34% at one, three and five years, respectively, with a median survival of 30 months (Figure 1). At last follow-up, 12 patients (44%) had died with disease. Of the 15 patients alive, nine (33%) were disease-free and six (22%) were alive with disease (two cases of hepatic disease, two with extrahepatic disease and two with both sites involved). Univariate analysis was performed with survival as an end point for all items concerning patient characteristics, data pertaining to the primary tumour (location, stage lymph node invasion, adjuvant chemotherapy, time interval between colectomy and hepatectomy), pre-operative chemotherapy (number of courses, number of lines of chemotherapy, utilization of chronomodulated therapy, type of regimen, response to chemotherapy), liver metastases (synchronous, bilobar versus unilobar, number, size, respectability, serum CEA and serum CA 19–9, metastatic pedicle lymph nodes), concomitant extrahepatic disease (location, curative resection) and technique of liver resection (utilization of portal embolization, two-stage versus synchronous procedures, combined radio-frequency ablation, major hepatectomy, anatomic versus non-anatomic resection, curative versus non-curative resection, number and size of metastases in the specimen, blood units transfused and duration of hospital stay). In some of these subgroups, there were very small numbers for adequate comparison.

**Figure 2:**
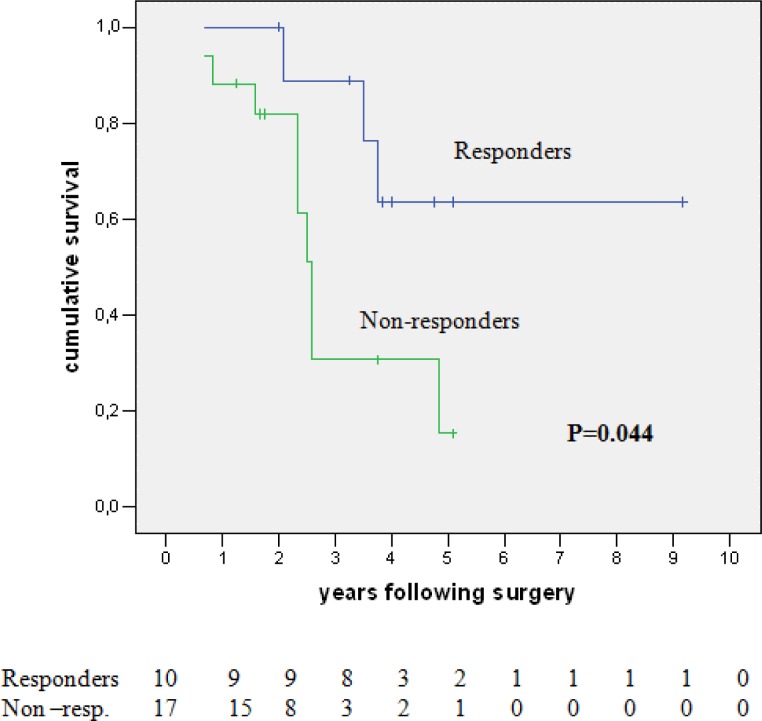
Survival of neoadjuvant chemotherapy patients—responders versus non-responders.

On univariate analysis, the only significant factor positively predicting survival was the response to neoadjuvant chemotherapy (64% versus 15% five-year survival for responder versus non-responders respectively, p=0.044). This effect is shown graphically in Figure 2. The response to neoadjuvant chemotherapy, the number of metastases and the total size of metastases, was matched in a multivariate analysis where the response to pre-operative chemotherapy was confirmed as the only significant prognostic variable affecting survival (HR 4.531; I.C. 1.002–21.204, p=0.05).

## Discussion

This small study shows that liver resection combined with pre- and post-operative (sandwich) chemotherapy offers the possibility of long-term survival of patients with chemoresponsive liver metastases (single/multiple and/or initially irresectable) from colorectal cancer. This benefit can be obtained only when the disease confined to the liver is controlled by chemotherapy prior to completely resectional surgery. Tumour progression whilst on pre-operative chemotherapy is associated with a poor outcome, even when hepatectomy is performed with curative intent.

This neoadjuvant approach has been reported to be associated with prolonged OS by others where it has been used for potentially resectable liver lesions, with demonstration of progression-free survival advantage over unresected cases [28]. It is, however, recognized that the global impact of sandwich pre- and post-operative chemotherapy on such progression-free survival is relatively low where, as found in our study, less than half of the cases resected are disease-free at five years [29]. Our approach does, however, appear safe with an acceptable morbidity, which is not exacerbated by the use of adjuvant chemotherapy [15,17] although post-operative hepatotoxicity is somewhat dependent upon the number of cycles administered rather than on the type of chemotherapy used [25]. Data concerning the hepatotoxicity of systemic neoadjuvant chemotherapy are, however, scarce [21] and may suggest a role in selected cases for interstitial concomitant therapies [30].

Available data would suggest that initial perioperative morbidity following hepatic resection appears to adversely affect the longer-term cancer-specific survival [31] but that medium-term health-related quality of life assessment after hepatectomy justifies such an aggressive approach [32]. There is no uniform policy regarding the role of synchronous over-staged hepatectomy in this setting. It would appear that although synchronous metastasis resection is safe and has oncologic merit, [33, 34] the potential benefit of the neoadjuvant approach is to define tumour biology where initial chemo-responsiveness selects those cases suitable for metastasis excision. This may be particularly evident when the primary tumour is more advanced, where overall cancer-specific prognosis is adversely affected when > 4 paracolic lymph nodes are involved [13].

It should be recognized that complete chemo-responsiveness on imaging may frequently be associated both with macroscopic and microscopic evidence of residual disease that may account for the relatively high incidence of hepatic in-situ recurrence found in our study as well as by others [35]. Tumour progression on chemotherapy is uniformly associated with a poor prognosis [36,37], as reported by Adam and colleagues, and similar to our study, this was found even when a potentially curative R0 hepatectomy was performed [38]. This group also noted (unlike our study) that the prognosis of radical hepatectomy after neoadjuvant therapy was affected by the number of metastases and the pre-operative serum level of CA 19-9, but similar to our findings, that the type of chemotherapy was not significant to the outcome provided that there was a therapeutic response. As in our study, others have reported that the presence of some types of extrahepatic disease does not contraindicate hepatectomy or subsequent excision of resectable extrahepatic sites in selected cases [39]. Where recurrence is restricted to the liver, as occurred in our study in one-third of recurrent cases, this may be amenable to repeat hepatic resection [40]. Outcome in this circumstance is dependent upon the radicality of re-resection in much the same way that it is in the initial resection.

In this study, the number of nodules was not a contraindication to resectional surgery. The five-year survival following such R0 liver resection was 34% in our cases: data that compare favourably with other surgical series reporting three-year and five-year survival of 21% [41] and 23%, respectively [33, 42]. One question that remains is whether the response to chemotherapy in our cases simply identifies patients who have a pre-determined favourable prognosis or whether the response is able to modify the actual course of the disease. Supporting the latter hypothesis, progression was clearly identified as an independent adverse prognostic factor of outcome on multivariate analysis. Moreover, treating resectable patients with neoadjuvant chemotherapy did not lead to non-resectable metastases during the course of the study: an effect also observed by others [29, 38]. This effect, combined with long-term survival in some cases initially defined as non-resectable which became down-staged by the neoadjuvant approach permitting complete resections, argues for the possibility that the course of the disease could be altered by such an aggressive strategy [24, 43, 44].

In conclusion, the response to neoadjuvant chemotherapy plays a key role in the potential benefit offered by radical liver resection in selected patients with hepatic metastatic disease from colorectal cancer. The poor results obtained by surgery in patients with tumour progression suggest that control of the disease with a modern combination regimen is preferable to immediate surgery. This delayed approach awaiting initial chemo-responsiveness might also prove to be relatively liver sparing and better defines those patients who are potentially advantaged by a formal hepatic resection as opposed to a tailored segmentectomy. Moreover, in those cases where larger metastases completely respond in one area of the liver, resection of the contralateral lobe may become an operative option [35]. This view is corroborated by recent evidence to show that the prognosis is adversely affected in those patients undergoing synchronous resection of hepatic metastatic disease with their primary tumour when the primary is extensive (T4), when it is infiltrating adjacent structures and when there are multiple hepatic metastases [45].

Overall, the data concerning neoadjuvant benefit to radical hepatectomy in colorectal hepatic metastatic disease are hard to interpret. This is as a result of variable histopathology in the liver, differing chemotherapy schedules, varying types of liver resections and different ischaemia/reperfusion cycling techniques during hepatic parenchymal transection. In this setting, it is not surprising that patients with multiple metastases tend to have more prolonged chemotherapy exposure, more extended resections and larger tumour/remnant liver ratios. Resections for hepatic colorectal metastases are increasing in incidence [46] and it is expected that the approach towards neoadjuvant therapy even for resectable hepatic metastases will be modified in the future by the concomitant use of anti-angiogenic therapy [47] followed by an aggressive approach.

## Figures and Tables

**Table 1:**
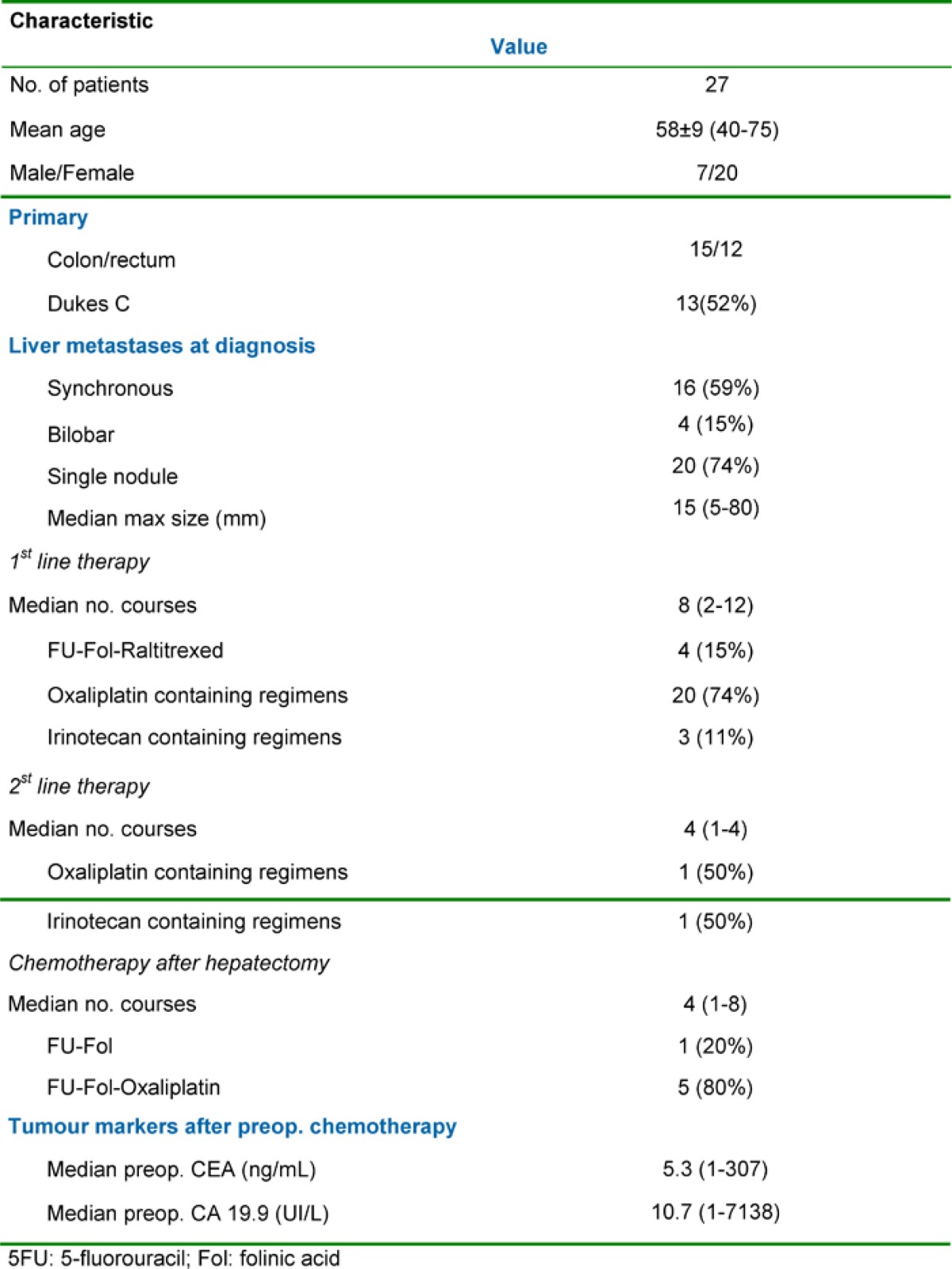
Patients, tumour characteristics and pre-operative chemotherapy

**Table 2:**
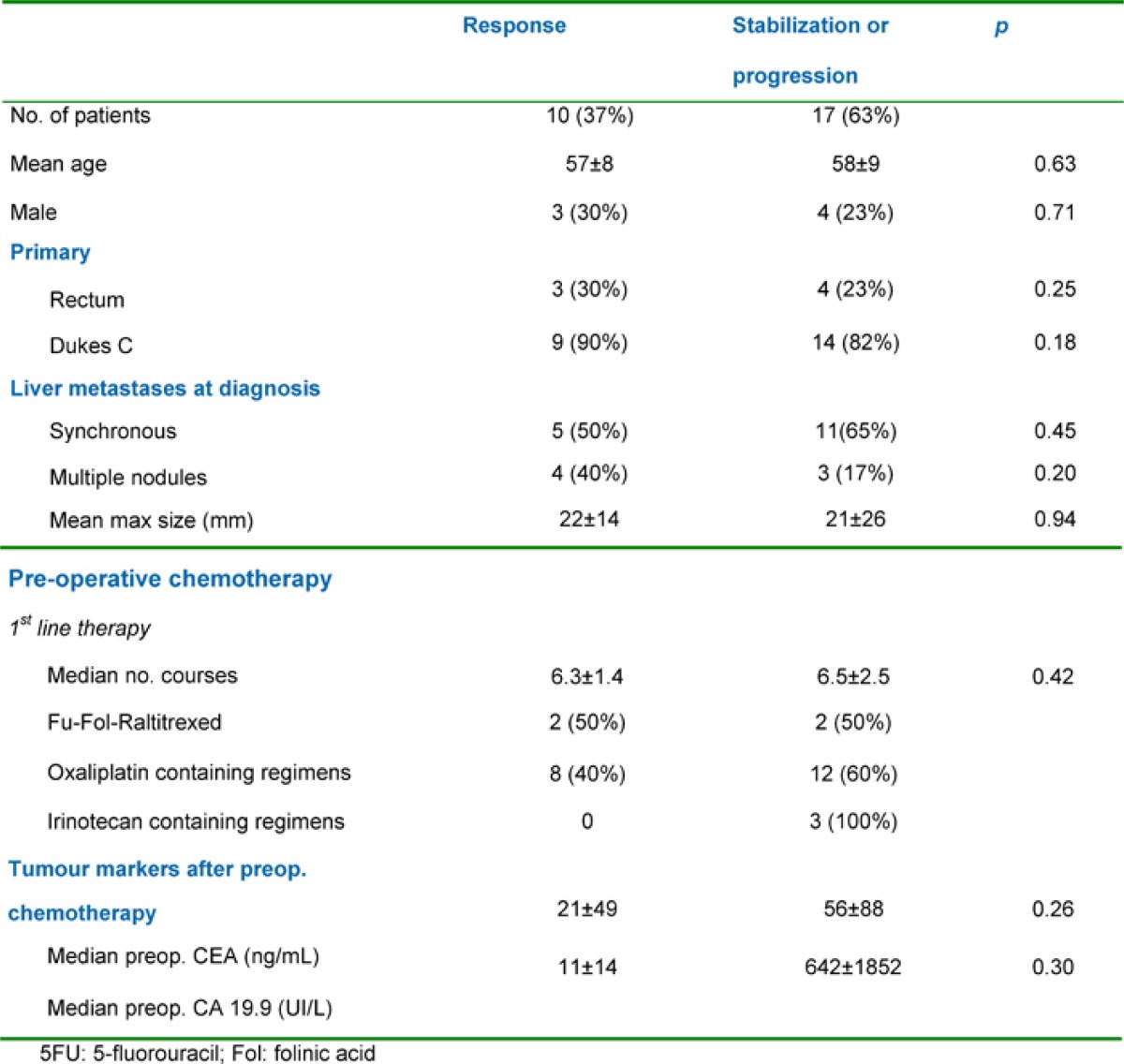
Patient and tumour characteristics according to response to pre-operative chemotherapy

## Abbreviations used in the text:

NACT: neoadjuvant chemotherapy
SD: stable disease
CT scan: computed tomography
MRI: magnetic resonance imaging
IOUS: intra-operative ultrasonography
CUSA: ultrasonic dissector-harmonic scalpel
TARF: radio-frequency ablation device
CVC: central venous catheter
